# The Lives We Have Bid to Be


**Published:** 1894-05-26

**Authors:** 


### STUDIES IN APPLIED SOCIOLOGY.

### IX.—"THE LIVES WE HAVE BID TO BE."*

The responsibility of a parent for his child begins at his birth—we mean the parent's birth, and not the child's. True it is that in the years of infancy and youth the responsibility is delegated to those who bring him up, but with manhood and womanhood, years before marriage, the responsibility becomes personal—it should become conscious. For generations past physicians have been protesting against the future mothers of the race pinching their waists and otherwise injuring their health; but how seldom do they speak words of advice to the future fathers. The reasons are simple enough. Women's folly is simply foolish, but the folly of men is immoral, to be condemned strenuously enough in private interviews with the physician, but not to be spoken of, like corsets and high heels, in journals meant for all. A reaction has set in. The new idea—which, after all, is not so new as it seems, for more than a generation ago Coventry Patmore praised the man "who was faithful to his future wife"—is promulgated far and wide. But the new idea fails because it is selfish, and, moreover, cuts both ways. To demand that a man be as pure as a woman may, in practice, end in the suggestion that a woman need be no purer than the average man. Indeed, looking to the success of "The Heavenly Twins" in fiction and "The Second Mrs. Tanqueray" in drama, may one not say that this has already largely come about? Paula is as sympathetic a character as Evadue; as likely, indeed, to affect the opinions and actions of the world.

But to put the question on so narrow a basis is an error. It is not the individual man or woman who is the social unit, but the family. Husbands and wives may be left to settle their own rights and wrongs so long as both unite in the due protection of their children's interests. And if they do this there will be little left to fight about. No State has ever prospered long where the sanctity of family life has not been honoured. The great age of Greece, rich as it was in statesmen, poets, and philosophers, lasted barely two hundred years. Is it too rash to attribute this to a state of public opinion which it made it possible for a Plato to suggest a community of wives as a feature of the ideal republic; to notions of life which made the wife only the drudge, the *hetaira* the intellectual companion of man? Rome, with infinitely less of genius than its neighbour of the East, holding strictly to the claim of the family even at the cost of the individual, became the mistress of the world, and left its laws to be the foundation of the jurisprudence of all time. But when under the empire the marriage tie became lax, Rome, too, declined and fell. The polygamy of Asia has been the fruitful source of crimes innumerable. A lady traveller tells that in India she was frequently asked by her patients for some potion to kill the co-wife or her children. From such risks the children of England are doubtless free, but can they escape from subtler and not less fatal evils? A reformed rake may, though it is doubtful, make the best husband, but assuredly he does not make the best father. In his offspring he will see the result of follies and vices indulged in years before their birth. Happy are the parents who see instead the result of a youth of self-restraint and purity. For good or evil the law of heredity exists, and cannot be evaded.

It is, of course, impossible to lay down one clear straight line of heredity. All the children of one family are not exactly alike. They "take after" each parent in varying proportions, and sometimes reproduce the half-forgotten traits of remoter ancestors. Any explanation of this must be more or less a matter of surmise, though the surmise can be based on sound enough reasoning. The old wives' tradition of attributing a child's characteristics to the thoughts and habits of his mother in the months preceding his birth has been accepted by scientists. It is more difficult to trace the pre-natal responsibility of the father, but it may reasonably be supposed to have an influence that cannot be ignored. Not merely habits of intoxication and sensuality, but fleeting indulgence, moments of weakness, may be reflected through the whole course of a life which in one of those moments has its mysterious beginning. The old query, "Did this man sin, or his father, that he was born blind?" though unjust in one recorded instance, is nevertheless reasonable in many cases, though not as the Jews imagined, because of the malice of a vindictive God, but because there is no condonation of an infringment of the laws of nature. The self-indulgent father, the frivolous mother, can hardly hope for wise and unselfish children. This is hardly realised yet, but when it is it will not be held less reprehensible for man or woman to waste the health of body and strength of mind which they wish their children to inherit, than to waste the money they should transmit to them.

The virtues of parenthood, however, like others, grow with use. And there is a regrettable tendency in modern sentiment to deprive parents of the wholesome discipline their position naturally provides for them. In our democratic desire to give every child the fairest possible start in life we may be depriving his parents of a divinely-appointed education in the higher virtues, and at the same time robbing the child of those ideas of responsibility from which in turn his children should profit. To throw all burdens on that vague incorporate entity called the State is to destroy those very qualities by which a State grows strong. Take away from a father the duty of providing food and education for his child, from a mother the task of watching over her child in sickness and in health, and you destroy the simple and natural steps by which they can hope to reach the higher notions of parental responsibility we have tried to indicate here.

* See "Marriage and Heredity," by Mrs. Joseph Lucas, British Women's Temperance Union. Price One Shilling. Also "The Family" in Bishop Westcott's "Social Aspects of Christianity." (Macmillan and Co.). Price 3s. 6d.

